# Silicon-Based All-Dielectric Metasurface on an Iron Garnet Film for Efficient Magneto-Optical Light Modulation in Near IR Range

**DOI:** 10.3390/nano11112926

**Published:** 2021-11-01

**Authors:** Denis M. Krichevsky, Shuang Xia, Mikhail P. Mandrik, Daria O. Ignatyeva, Lei Bi, Vladimir I. Belotelov

**Affiliations:** 1 Moscow Institute of Physics and Technology (MIPT), 141700 Dolgoprudny, Russia; 2 Russian Quantum Center, 121353 Moscow, Russia; daria.ignatyeva@gmail.com (D.O.I.); belotelov@physics.msu.ru (V.I.B.); 3 Physics and Technology Institute, Vernadsky Crimean Federal University, 295007 Simferopol, Russia; 4 National Engineering Research Center of Electromagnetic Radiation Control Materials, University of Electronic Science and Technology of China, Chengdu 610054, China; xiashuang_1996@163.com (S.X.); bilei@mit.edu (L.B.); 5 State Key Laboratory of Electronic Thin-Films and Integrated Devices, University of Electronic Science and Technology of China, Chengdu 610054, China; 6 Faculty of Fundamental Physical and Chemical Engineering, Lomonosov Moscow State University, 119991 Moscow, Russia; skyworker14@yandex.ru; 7 Photonic and Quantum Technologies School, Lomonosov Moscow State University, 119991 Moscow, Russia

**Keywords:** magneto-optics, dielectric nanophotonics, transverse magnetooptical Kerr effect

## Abstract

All-dielectric nanostructures provide a unique low-loss platform for efficiently increasing light-matter interaction via excitation of the localized or propagating optical modes. Here, we report on the transverse magneto-optical Kerr effect enhancement in an all-dielectric metasurface based on a two-dimensional array of Si nanodisks on a cerium substituted dysprosium iron garnet thin film. We observed up to 15% light intensity modulation under TM modes excitation. The observed magneto-optical effect is nearly independent of the rotation of the light incidence plane with respect to the metasurface. Being compatible with conventional semiconductor technology, our structure holds promise for device applications, such as light modulators, magnetic and chemical sensors.

## 1. Introduction

Light manipulation via nanostructured materials is a prominent issue of modern nanophotonics. The advances of nanophotonics are currently applied in various technical fields, such as information processing [1,2,3,4,5,6], physical and chemical sensing [7,8,9,10,11], and quantum simulations [12]. Magnetic materials provide additional degree of freedom for light control via various magneto-optical effects, such as Faraday, Voight, transverse and polar Kerr effects [13]. Excitation of various optical modes, such as surface or localized plasmons [14,15,16,17,18,19,20,21], waveguiding modes [22,23,24,25,26], surface Tamm states, and cavity modes in photonic crystals [9,10,27,28] strongly enhance the aforementioned effects in magnetic nanostructures. Metal nanostructures produce a notable field localization and enhancement in the metal based magnetic nanostructures. At the same time, light-matter interaction in such systems is always accompanied by high optical losses and heating, making them less suitable for device applications. At this point, all-dielectric nanostructures are very promising since they are substantially less dissipative.

Dielectric iron oxides, for instance, iron garnet thin films and crystals, are widely used in magneto-optical devices. Magneto-optical properties of these compounds strongly depend on elemental composition [13]. All-dielectric iron-garnet nanogratings were reported to boost the magneto-optical response [22,23]. However, the fabrication of iron garnet-based nanostructures, such as one- or two-dimensional gratings, is a complicated process that is typically accompanied by focused ion beam (FIB) technology [22,23]. A dielectric grating is used to couple light with matter and excite optical modes. For these purposes, widely used semiconductor materials can be utilized, such as GaP, GaAs, InP, and Si. The latter material has a well-developed technological process of deposition, processing, and nanofabrication. Si-based nanostructures are currently used in a variety of applications, including chemical sensing [29], holography [30], flat optics [31], and data processing via light control (light wavelength, polarization state, transmission, and reflectivity) [32]. The mentioned technological advances make the combination of Si nanostructures with iron-garnet films an excellent candidate for enhanced magneto-optics.

In this paper, we report on an enhanced magneto-optical response observed in the all-dielectric structure based on a two-dimensional (2D) grating of Si nanodisks on a cerium substituted dysprosium iron garnet thin film in the near IR range. The periodicity of grating allows the excitation of the guided modes in the magnetic layer, which mediates a resonant increase of the transverse magneto-optical Kerr effect (TMOKE). TMOKE amplitude and spectral position are shown to be almost independent of the sample rotation around its normal. This feature, combined with the ease of fabrication process, makes the structure promising for applications in sensing and magnetometry.

## 2. Materials and Methods

### 2.1. Samples Fabrication

Pulsed laser deposition (PLD) was used to grow a 150 nm thick cerium substituted dysprosium iron garnet thin film of composition (Ce_1_Dy_2_)(Al_0.42_Fe_4.58_)O_12_ (Ce:DyIG) with a 50 nm thick yttrium iron garnet (YIG) layer on a fused quartz substrate. The targets were ablated with a 10 Hz, 248 nm KrF excimer laser. The 50-nm-thick YIG film was first deposited on the silica substrate and served as a seed layer to promote the crystallization of the upper Ce:DyIG film. The substrate temperature was 400 °C and oxygen pressure was 10 mTorr during the YIG deposition process. The film was then rapidly annealed for 480 s at 900 °C and 80 Torr oxygen pressure. The aluminum-doped 150-nm-thick Сe:DyIG was deposited by exchanging targets of Ce_1_Dy_2_Fe_5_O_12_ and Ce_1_Dy_2_Al_1_Fe_4_O_12_ at the substrate temperature of 750 °C and an oxygen pressure of 5 mTorr.

**Figure 1:**
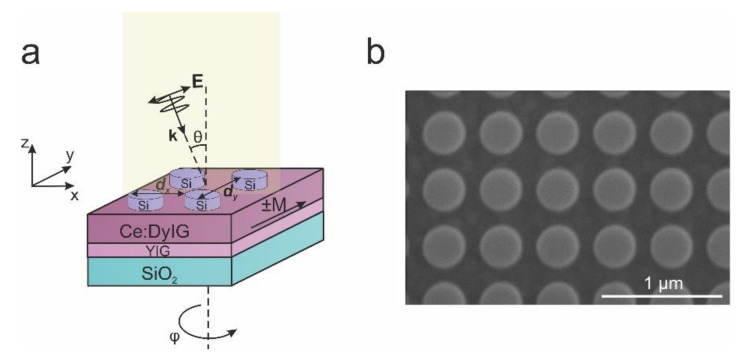
Schematic representation of the magneto-optical metasurface of Si nanodisk array on a Ce:DyIG (**a**) and SEM image of the sample (**b**).

Following the deposition of the magneto-optical films, an amorphous silicon thin film of 120 nm thickness was grown via plasma-enhanced chemical vapor deposition (PECVD). The patterns of a negative electron-beam resist HSQ were then exposed using electron beam lithography (EBL). Following that, a two-dimensional array of the Si nanodisks of 170 nm radius was fabricated using reactive ion etching (RIE) with HSQ as the resist. The Si nanodisks form a grating with a square lattice and a 500 nm period (Figure 1).

### 2.2. TMOKE Measurements

The angle-resolved transmittance and TMOKE spectra were measured using a Fourier experimental setup. The light from a halogen lamp (spectral range from 360 to 2500 nm) was collimated with a lens (focal length 35 mm) and polarized with a Glan–Taylor prism. Linearly polarized light after the Glan–Taylor prism was focused on the sample using a 20× microscope objective. The p-polarized (polarized in the incidence plane) light passed through the sample was collimated on the spectrometer by another 20× microscope objective and a system of lenses with focal length 300 mm and 150 mm. The transmittance was obtained by comparing the sample’s spectrum to the spectrum of the light source. For angle-resolved TMOKE spectra measurements, the sample was placed in a uniform magnetic field of 100 mT generated by an electromagnet in transversal configuration (see Figure 1a). TMOKE dependence on the azimuth angle was also measured by rotating the sample around its normal. In this case, the transversal configuration of the magnetic field with respect to the light incident plane was preserved. The scheme of the setup is presented in Appendix D (see, Figure A4).

### 2.3. Numerical Simulation

**Figure A1:**
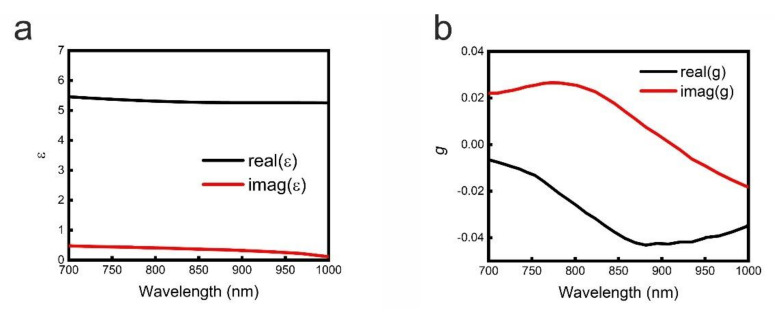
Dielectric permittivity (**a**) and gyration (**b**) of Ce:DyIG.

The rigorous coupled-wave analysis (RCWA) approach was utilized for electromagnetic numerical simulation of the structure’s optical and magneto-optical properties [33,34]. For simplicity, Dy:CeIG and YIG layers were substituted by a single 200 nm thick magnetic layer with the components of dielectric permittivity tensor given in Appendix A (Figure A1). It was deduced from the optical spectra of the magnetic sample without Si nanodisks. The dielectric permittivity of Si was taken from reference [35]. The dielectric permittivity of the glass substrate was 2.10.

## 3. Results and Discussions

### 3.1. Optical Modes

**Figure 2:**
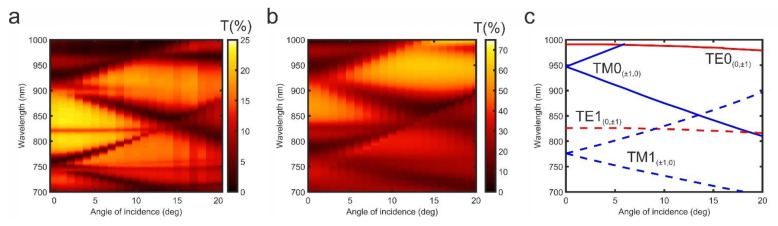
Angle resolved transmission spectra of the sample under p-polarized light excitation. (**a**) Experimental, (**b**) numerical, (**c**) calculated modes dispersion.

Experimentally measured transmission spectra of the sample contain a number of well-defined resonances (Figure 2a). The transmission spectrum of the sample exhibits pronounced dips at ~985, 935, 828, and 768 nm at normal incidence of light. The resonances at 935 and 768 nm possess clear V-shaped angle-dependent evolution behavior as the polar incident angle (*θ*) increases. Simultaneously, resonances at 985 nm and 828 nm are almost independent of the polar angle.

The propagating guided optical modes drive the optical and magneto-optical properties of the examined metasurface. To confirm it, we first consider excitation conditions and dispersions for both TM and TE guided modes. The phase-matching condition must be met in order to couple incident light with matter via diffraction on Si nanograting:

(1) $$\beta^{2}={\left(k_{0}\sin\left(\theta \right)+2\pi \frac{m}{d_{x}}\right)}^{2}+{\left(2\pi \frac{n}{d_{y}}\right)}^{2}.$$

In Equation (1), β is the propagation constant of a mode, $k_{0}=\frac{2\pi }{\lambda }$ is the free space light wavenumber, *λ* is the free space light wavelength, θ is the polar incident angle, $d_{x},\;d_{y}$ are periods of the structure along the OX and OY directions correspondingly, and *m* and *n* are integers which represent the mode order along the OX and OY directions. The propagation constant β of the TM or TE guided modes can be calculated using the transcendental equation [36]:

(2) $$-p_{\left\{2,N\right\}}d+\tan^{-1}\left\{{\left(\frac{\epsilon_{2}}{\epsilon_{1}}\right)}^{r}\;\frac{p_{\left\{1,N\right\}}}{p_{\left\{2,N\right\}}}\right\}+\tan^{-1}\left\{{\left(\frac{\epsilon_{2}}{\epsilon_{3}}\right)}^{r}\;\frac{p_{\left\{3,N\right\}}}{p_{\left\{2,N\right\}}}\right\}=-N\pi ,\;r=\left\{\begin{array}{c} 0\;for\;TE\;modes\\ 1\;for\;TM\;modes \end{array}\right.,$$

where $p_{\left\{1,N\right\}}={\left(\beta^{2}-\epsilon_{1}k_{0}^{2}\right)}^{\frac{1}{2}}$, $p_{\left\{2,N\right\}}={\left(\epsilon_{2}k_{0}^{2}-\beta^{2}\right)}^{\frac{1}{2}}$, $p_{\left\{3,N\right\}}={\left(\beta^{2}-\epsilon_{3}k_{0}^{2}\right)}^{\frac{1}{2}}$, and $\epsilon_{j}^{}$ are dielectric permittivity of the iron garnet film ($\epsilon_{2})$ and surrounding claddings ($\epsilon_{1},\epsilon_{3}$), *N* is the integer that defines the order of the mode (along the OZ direction), and *d* is the core thickness. In the case of transversal magnetic configuration, Equation (2) does not change for *TE modes*, but modifies for *TM modes*:

(3) $$-p_{\left\{2,N\right\}}d+\tan^{-1}\left\{{\left(\frac{\epsilon_{2}}{p_{\left\{2,N\right\}}}\right)}^{}\left(\;\frac{p_{\left\{1,N\right\}}}{\epsilon_{1}}+\frac{g\cdot \beta }{\epsilon_{2}^{2}}\right)\right\}+\tan^{-1}\left\{{\left(\frac{\epsilon_{2}}{p_{\left\{2,N\right\}}}\right)}^{}\;\left(\;\frac{p_{\left\{3,N\right\}}}{\epsilon_{3}}-\frac{g\cdot \beta }{\epsilon_{2}^{2}}\right)\right\}=-N\pi ,$$

where *g* is a core material gyration constant proportional to its magnetization M.

We calculated the dispersion relation of the modes using Equations (1)–(3). Resonances in the 700–1000 nm spectral region correspond to both TE and TM guided modes (Figure 2c). As previously observed, the TE0_(0, ±1)_ and TE1_(0, ±1)_ (further TE0 and TE1) modes exhibit a weak dependence on incidence angle. On the contrary, resonance positions in transmission spectra of the TM0_(±1, 0)_ and TM1_(±1, 0)_ (further TM0 and TM1) modes are strongly influenced by *θ*. Notably, the TM0 and TM1 modes spectrally overlap at 850 nm and 14° incident angle. The angle-dependent transmittance spectrum simulated numerically using the RCWA method agrees well with the one obtained experimentally (Figure 2b). However, there are minor discrepancies between the calculated positions of the resonances and the ones obtained from experimental data in both transmission and TMOKE spectra. They are caused by the fabrication inaccuracies, which result in a slight difference between geometrical parameters (such as Ce:DyIG thickness and grating period) of the experimental metasurface with their calculated counterparts. Table 1 provides a brief summary of the revealed spectral position features of the resonances.

**Figure 3:**
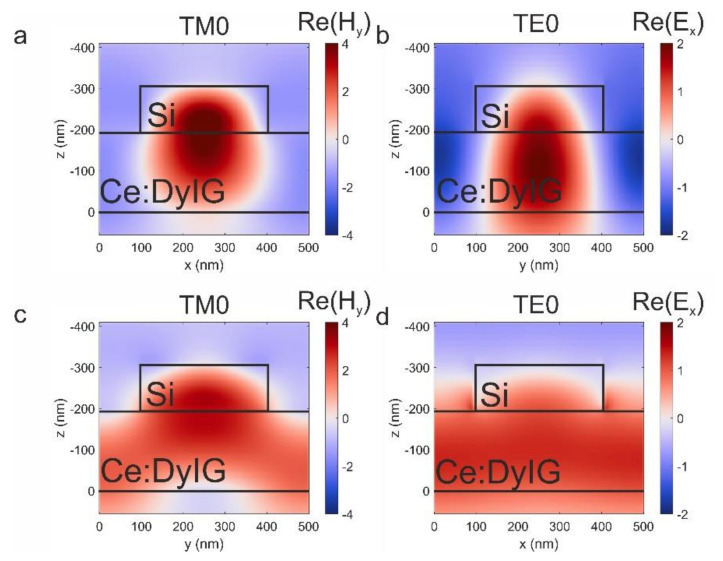
Electromagnetic field distribution of the TM0_(±1, 0)_ (**a**,**c**) and TE0_(0, ±1)_ (**b**,**d**) modes.

Electromagnetic energy of the waveguided modes is known to be concentrated inside the core. We numerically simulated the electromagnetic field distribution of optical modes excited by normally incident linearly polarized light to confirm the origin of the resonances. The TM(TE) guided modes possess elliptical polarization with nonzero E_x_(H_x_), E_z_(H_z_), and H_y_(E_y_) components. The TM0 guided mode induced by p-polarized light has nonuniform alternate sign *H_y_* and *E_x_* component distribution along the OX direction and uniform along the OY direction. The situation is inverse for the TE0 one (Figure 3b). There is no alternating sign field behavior along the OZ direction for both TE0 and TM0 modes.

Notably, the TE0 mode electromagnetic field is mainly concentrated inside the garnet film. However, in the TM0 case, the electromagnetic field is slightly squeezed into Si nanodisk. As a result, the metasurface should be considered as a complex nonuniform waveguide. Furthermore, each Si nanodisk also serves as a scatterer allowing optical and magnetooptical features of the system to be detected in the far field.

**Figure A2:**
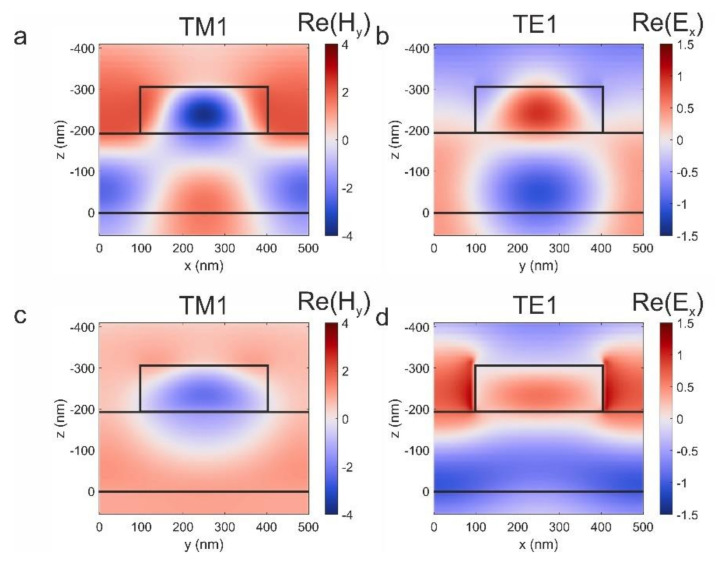
Electromagnetic field distribution of the TM1_(±1, 0)_ (**a**,**c**) and TE1_(0, ±1)_ (**b**,**d**) modes.

The electromagnetic field distribution of the TM1 and TE1 modes along the OX and OY directions is similar to the behavior of TM0 and TE0 modes (see Appendix B, Figure A2). The primary discrepancy is observed along the OZ direction. While the electromagnetic field distribution of the TM0/TE0 modes is nearly uniform, the TM1/TE1 modes have two antinodes that correspond to the order of the modes.

### 3.2. TMOKE Boosted by the Waveguiding Modes

The transverse magneto-optical Kerr effect (TMOKE) is the magneto-optical intensity effect that can be calculated as follows:

(4) $$\delta =2\frac{T\left(+M\right)-T\left(-M\right)}{T\left(+M\right)+T\left(-M\right)}\times 100\%$$

where *T*(±*M*) denotes the sample transmittance under antiparallel orientations of the magnetic field (namely, +*M* and −*M*) in the transversal configuration, i.e., for an external magnetic field applied perpendicular to the light incidence plane. TMOKE is typically observed in gyrotropic materials with dissipation [13]. However, in the optical range where iron garnets are relatively transparent for a smooth film, δ is rarely greater than ~10^−2^%.

**Figure 4:**
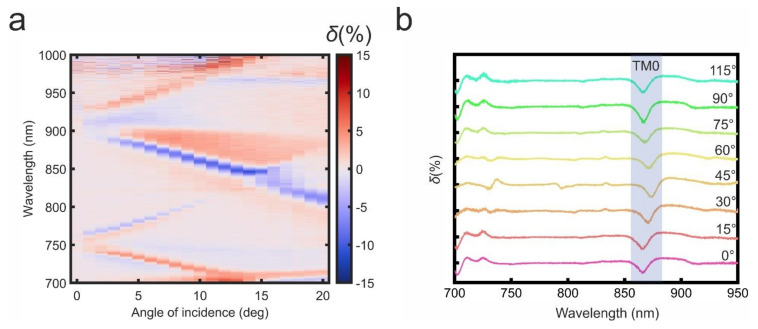
Experimental TMOKE spectra as a function of *θ* (**a**) and for a fixed polar incident angle *θ* = 10° as a function of the azimuth incident angle *φ* (**b**). All curves in (**b**) have offsets to clarify the representation.

**Figure A3:**
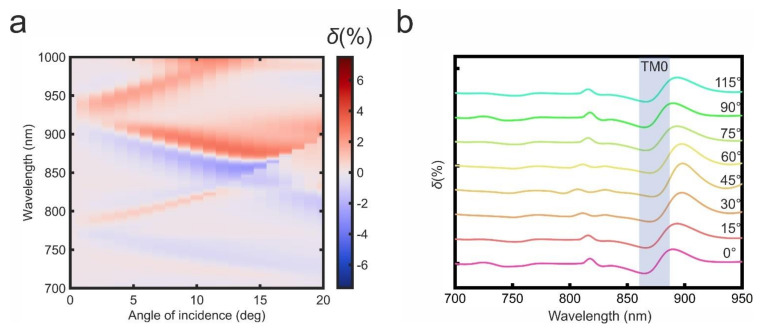
Numerically simulated TMOKE spectra as a function of θ (**a**) and for fixed θ = 10° as a function of the sample rotation angle φ (**b**). All curves in (**b**) have offsets to clarify representation.

**Figure A4:**
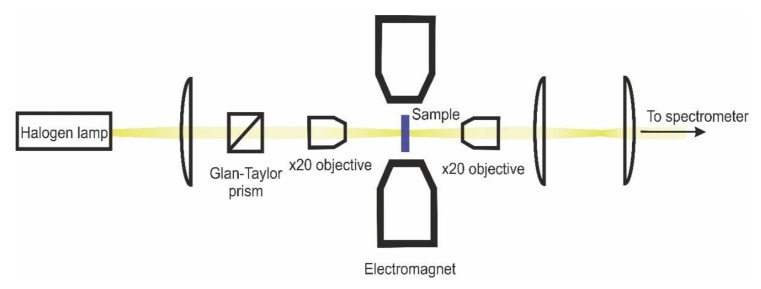
Schematic of TMOKE setup used in the experiment.

In the transversal magnetization (*M*) configuration the TM guided modes propagating constant is contributed to by an additional component proportional to magnetization $\beta_{TM}=\beta_{TM}^{0}+\Delta \beta_{TM}\left(M\right)$ (see Equation (3)). This nonreciprocal contribution to the propagation constant influences transmission spectra, resulting in sharp U-shaped resonances in the TMOKE spectra measured experimentally (Figure 4a). TMOKE spectra calculated numerically match those obtained experimentally (see Appendix C, Figure A3). The Q-factor ($\frac{\lambda }{\Delta \lambda }$) of the TMOKE resonance reaches 85.

TMOKE is significantly enhanced in the metasurface under the excitation of TM0 and TM1 modes (Figure 2c and Figure 4a). The greatest δ increase up to 14% is observed under the TM0 mode excitation in the angular range *θ* = 5 ÷ 15° for the wavelengths 850–890 nm (Figure 4a). This value is about three orders of magnitude larger in comparison to a pristine Ce:DyIG film of the same thickness.

TMOKE potential independence from the orientation of the plane of light incidence with respect to the metasurface is highly demanded for many applications, such as optical modulators, sensors, and magnetometers. Guided by this idea we studied the TMOKE dependence on the azimuth incident angle. We concentrated on the TM0 mode excited at ~860 nm under 10° polar incident angle where a pronounced TMOKE enhancement occurs (Figure 4b). Notably, the TMOKE resonance has only a minor spectral deviation when mediated by the TM0 mode, making the proposed magneto-optical metasurface promising for light modulation devices and other applications.

## 4. Conclusions

We designed and fabricated a magneto-optical metasurface for efficient light control via an external magnetic field. It was shown experimentally and numerically that TMOKE is enhanced by about three orders of magnitude in comparison to the pristine Ce:DyIG film due to the TM guided modes excitation. It is critical to note that the TMOKE resonances are unaffected by the orientation of the light incidence plane in relation to the metasurface. The latter feature is important for device applications such as light modulators. Biosensing is another potential application for the suggested gadget. Sharp and prominent resonances of TMOKE may be highly sensitive to the refractive index of the adjacent medium allowing the metasurface to provide sensing functionality. The latter is to be examined in detail in the future. Moreover, as the structure grating is made of Si, the production process is flexible and inclusive of current semiconductor technology.

## Figures and Tables

**Table 1:** Guided modes’ resonant wavelength observed in the transmission spectra.

Waveguide Mode	Diffraction Order (m, n)	Resonant Wavelength from Experiment (nm)	Resonant Wavelength from Simulation (nm)	Resonant Wavelength from Equations (1)–(3) (nm)
TE0	(0, ±1)	985	1000	991
TM0	(±1, 0)	935	950	947
TE1	(0, ±1)	828	933	826
TM1	(±1, 0)	768	788	776

## Data Availability

The data presented in this study are available on request from the corresponding author.
